# 532 nm Low-Power Laser Irradiation Facilitates the Migration of GABAergic Neural Stem/Progenitor Cells in Mouse Neocortex

**DOI:** 10.1371/journal.pone.0123833

**Published:** 2015-04-28

**Authors:** Yumi Fukuzaki, Hyeryun Shin, Hideki D. Kawai, Banri Yamanoha, Shinichi Kogure

**Affiliations:** 1 Department of Bioinformatics, Graduate School of Engineering, Soka University, Hachioji, Tokyo, Japan; 2 Department of Environmental Engineering for Symbiosis, Faculty of Engineering, Soka University, Hachioji, Tokyo, Japan; Ajou University, REPUBLIC OF KOREA

## Abstract

**Background and Objective:**

Accumulating evidence has shown that low-power laser irradiation (LLI) affects cell proliferation and survival, but little is known about LLI effects on neural stem/progenitor cells (NSPCs). Here we investigate whether transcranial 532 nm LLI affects NSPCs in adult murine neocortex and in neurospheres from embryonic mice.

**Study Design/Materials and Methods:**

We applied 532 nm LLI (Nd:YVO_4_, CW, 60 mW) on neocortical surface via cranium in adult mice and on cultured cells from embryonic mouse brains in vitro to investigate the proliferation and migration of NSPCs and Akt expression using immunohistochemical assays and Western blotting techniques.

**Results:**

In vivo experiments demonstrated that 532 nm LLI significantly facilitated the migration of GABAergic NSPCs that were induced to proliferate in layer 1 by mild ischemia. In vitro experiments using GABAergic NSPCs derived from embryonic day 14 ganglionic eminence demonstrated that 532 nm LLI for 60 min promoted the migration of GAD67-immunopositive NSPCs with a significant increase of Akt expression. Meanwhile, the LLI induced proliferation, but not migration, of NSPCs that give rise to excitatory neurons.

**Conclusion:**

It is concluded that 532 nm LLI promoted the migration of GABAergic NSPCs into deeper layers of the neocortex in vivo by elevating Akt expression.

## Introduction

Since Mester’s first report which demonstrated the improvement of intractable skin ulcer by low-power laser irradiation (LLI) [1], there are many studies showing LLI-mediated cell proliferation and survival in various fields [2–6]. Those studies have been performed using red to infra-red LLIs because of their penetration property into relatively deeper sites [7]. In cell culture systems using other wavelength LLIs, we can find reports showing that 532 nm LLI promoted proliferation of B-14 (Chinese hamster ovarian cell line) cells without inducing cell death [8], it influenced blood platelets to trigger signal transduction, leading to platelet activation [9], and it significantly increased cell survival of human adipose tissue-derived stem cells following mitochondria activation [10].

LLI has been examined for its potential in the treatment of brain diseases. It has been reported that transcranial LLI promotes recovery from ischemic stroke, traumatic brain injury, and neurodegenerative diseases [11]. Yip et al. demonstrated that 660 nm LLI decreased the expression of pro-apoptotic factors (caspase 3 and caspase 9) while increased expression of antiapoptotic factors, such as Akt, pAkt, Bcl-2 and pBAD, following transient cerebral ischemia [12]. Oron et al. reported 880 nm LLI decreased neurological deficits and increased neurogenesis in the subventricular zone after acute stroke by permanent middle cerebral artery occlusion in rats [13]. Recently, 808 nm LLI therapy showed initial safety and effectiveness choice as a new treatment strategy for human ischemic stroke [14]. Zhang et al. showed that 810 nm LLI not only inhibited inflammatory mediators generated by gentle traumatic brain injury but also dramatically inhibited secondary brain injury in mice lacking immediate early response gene X-1 [15]. These reports suggest that red or near infra-red LLI has anti-apoptotic and anti-inflammatory effects, and that it could delay the progress of neurological diseases. However, there has been no report regarding effects of LLI on neurogenesis of neural stem/projenitor cells (NSPCs).

We have previously demonstrated that infra-red 808 nm LLI delayed cell cycle and suppressed the cell proliferation of human-derived glioblastoma A-172 [16] and 405 nm LLI promoted the cell death of A-172 cells [17], but 532 nm LLI promoted cell proliferation via Akt activation [18]. The latter phenomenon is consistent with other reports which showed that 632.8 nm LLI prevented apoptosis via Akt activation, although the different wavelength of LLI was used [19, 20].

It is well known that activated Akt plays key roles in mediating cell proliferation, cell survival (anti-apoptotic), cell-cycle progression, differentiation, transcription, translation, and glucose metabolism. In NSPCs, the elevating Akt activity in vitro increased cell survival and proliferation in cultured NSPCs derived from embryonic day 13 (E13) mouse cortex [21], whereas Akt activation induced cell differentiation in cultured NSPCs derived from the medial ganglionic eminence (MGE) of E14 mouse [22]. Akt signaling cascades are also stimulated almost exclusively in actively proliferating NSPCs in the adult hippocampus [23].

The hippocampal subgranular zone (SGZ) and the forebrain subventricular zone (SVZ) are well known as the places where adult neurogenesis occurs. Although spontaneous adult neurogenesis is not yet known to occur in cerebral cortex, cortical neurogenesis may be induced by mild ischemia in MGE-derived NSPCs present in layer 1 of adult rat cortex, where NSPCs migrate to deep layers, possibly contributing to changes in neural circuits [24]. Since we are not aware of any published reports of LLI effects on NSPCs, we investigated whether transcranial 532 nm LLI affects NSPCs in adult murine neocortex as well as in cultured NSPCs from embryonic mice.

## Materials and Methods

We performed all experiments in accordance with the Declaration of Helsinki and the Guidelines of Animal Use Committee at Soka University. The name of Committee which approved this study plan is The Soka Bioethics Committee for Life-science. Animals were housed in facilities with a 12/12 h light/dark cycle. For in vivo or in vitro experiments, the adequate procedures including anesthetics and methods of sacrifice so as to make the suffering at the lowest level were approved by the Committee and described in each section.

### Laser Irradiation Method

**Fig 1:**
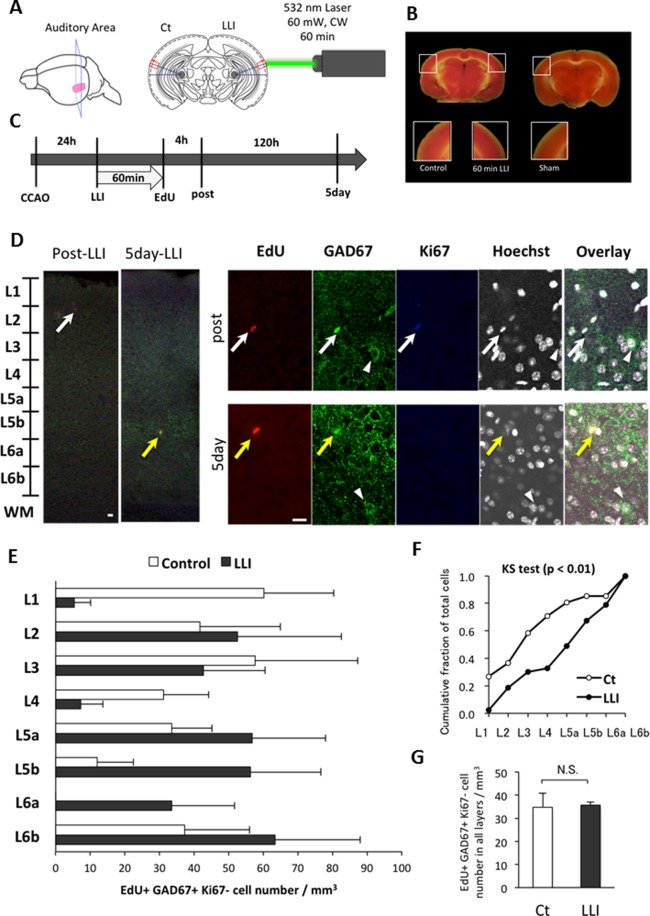
Effects of transcranial LLI on cell migration of NSPCs. A: Scheme of LLI treatment to adult mouse brain. Auditory cortical area was irradiated on the left side of the cortex through cranium. The opposite hemisphere was used as a control. B: TTC staining for detecting cell death. LLI after the mild occlusion of CCA (CCAO) did not induce cell death (left) compared with sham control (right). C: Protocol for in vivo experiments from CCAO to immunostaining. Brains were fixed at 4 h and 5 days after LLI. D: Photographs of each immunostaining at post-LLI (4 hours) and 5 days after LLI. EdU, GAD67 and Ki67 positive cells (white arrow) were found in layer 1 of post-LLI sections. EdU and GAD67 positive cells but Ki67 negative cells (yellow arrow) were found in deep layer at 5 days after LLI. GAD67 positive, EdU and Ki67 negative cell (arrowhead) is a mature inhibitory neuron. Scale bars: 10 μm. E: The laminar pattern of the cell density (mean ± SEM, n = 4) of EdU and GAD67 positive, Ki67-negative cells from layer 1 to 6 was significantly different between LLI and control groups (KS test, p<0.01). F: Cumulative fraction of total cells for non-parametric KS 2-sample test. Each accumulative curve shows higher distribution in layer 1–4 in control group, while does in layer 5–6 in LLI group. G: Total cell density of EdU and GAD67 positive and Ki67 negative (EdU+/GAD67+/Ki67-) cells did not differ between control and LLI groups.

A diode laser apparatus (Nd:YVO_4_, CW, 532 nm, 0–180 mW: SUWTECH, LDC-2500, China) was used in all experiments. The power measured in front of irradiated cortical surface was 60 mW on average and the irradiated area was 7.1 mm^2^; thus the power density was 845 mW/cm^2^. Irradiation time was 60 min for experiments in vivo and 20, 40 and 60 min for experiments in vitro with an energy density of 10.1, 20.2, 30.3×10^2^ J/cm^2^, respectively. For in vivo experiments, FVB mice were anesthetized with a mixture of nitrous oxide and oxygen gas (2:3), and fixed on a stereotaxic apparatus. LLI was made perpendicular to the surface of the temporal skull over the left auditory cortex (Fig 1A), while non-irradiated right auditory cortex was used as a control. The same laser irradiation method for in vitro experiments described previously [18] was used.

### Transient mild ischemia

Adult FVB mice (>60 postnatal days) were anesthetized with N_2_O/O_2_ gas, placed on a heating pad with supine position. A midline of the neck was opened, and common carotid artery (CCA) was separated from the surrounding tissue. Then bilateral CCAs were occluded by floss for 10 min (experimental group) or not occluded (sham control group). After suturing, 2% lidocaine was applied, and waited until awakening.

### Triphenyltetrazolium chloride (TTC) staining

TTC (triphenyltetrazolium chloride, Wako) staining was used to detect cell death. Briefly, mice were decapitated after anesthetized with halothane. Brain was immediately taken out from the skull and placed into an iced PBS for 15 min. Brain was sliced in 2 mm thickness by a brain slicer on ice. Brain slices were incubated in 2% TTC in PBS at 37°C for 10 min. 10% formaldehyde was used in order to fix the stained brain slices.

### Neural Stem/Progenitor Cell (NSPC) primary culture

NSPC primary culture was performed according to the neurosphere methods previously described [25]. During the development of mouse cerebral cortex, the progenitor amplification occurs from E9 and neurogenesis starts from E11. After E18, the genesis of astrocytes and oligodendrocytes occurs along with maturation of neurons [26, 27]. Therefore, primary NSPCs were prepared from the MGE or cortical cerebral wall of E10.5, E14.5 and E16.5 mouse forebrain. Pregnant mice were sacrificed by cervical dislocation, embryos were removed, and their brains were dissected in PBS on ice. The brain tissue from each embryo was incubated in 2.5% trypsin for 30 min at 37°C water bath and then transferred to culture medium consisting of DMEM/F12 (1:1), supplemented with 100 μg/ml transferrin, 30 nmol/ml selenium, 10 μg/ml heparin, 25 μg/ml insulin, and 20 ng/ml basic fibroblast growth factor. The cells were cultured at a density of 1 x 10^5^ cells/ml in uncoated plastic 35 mm dish. The next day, suspension cells along with the medium was placed in a new dish to exclude differentiated cells adhered to the bottom of the dish, and the same volume of new medium was mixed. After NSPCs had been cultured for 3 days, resulting in forming neurospheres, each neurosphere was transferred to 96-well plates with round-bottom (Sumitomo Bakelite Co. Ltd., Tokyo, Japan), and then was used for LLI experiment. When neurospheres were transferred under the microscope by a pipette, we selected those that are non-adherent and sphere-shaped as NSPCs.

### Proliferation analysis and migration analysis in culture

Cell proliferation rate was measured by Cell Counting Kit-8 (CCK-8) colorimetric assay (Dojindo Co. Tokyo, Japan). At 24 or 48 hours after LLI, 10 μl of the CCK-8 solution was added to each well. The cells were incubated at 37°C for 4 hours. The absorbance at 450 nm was measured using a microplate reader (model 680, BIO RAD, Tokyo, Japan). Neurospheres were photographed with a microscope (BZ-9000, KEYENCE, Tokyo, Japan), and then single cells obtained from trituration of neurospheres were stained with DAPI and photographed.

For migration assay, polycarbonate membrane inserts with 8 μm pore size (Corning Transwell: Sigma) was used. After LLI, cells on the lower side of the insert filter were fixed with 4% paraformaldehyde for 40 min, permeabilized with 0.1% Triton X-100 for 5 min, and preincubated with blocking solution (1% bovine serum albumin, 1% goat serum in PBS) followed by a primary antibody against GAD67 (1:1000, Millipore) incubation overnight at 4°C and a secondary antibody (Rhodamine- or FITC-conjugated) incubation for 3 hours under room temperature. Cells were stained with Hoechst 33342 (Sigma) for 20 min. The number of cells on the lower side of the filter was counted under a microscope. Images were analyzed using an optional software for neurosphere size and the number of nuclei.

### Immunohistochemistry

For the analysis with 5-ethynyl-2’-deoxyuridine (EdU), animals were intraperitoneally injected with EdU (50 mg/kg mouse) once after LLI. At 4 hours or 5 days after the LLI, animals were deeply anesthetized with urethane (1.2 g/kg: Sigma) and xylazine (13 mg/kg: Sigma), and sacrificed by perfusion with ice-cold PBS and 4% PFA. Brain was removed and post-fixed in 4% PFA for 2 hours at 4°C. After washing with PBS, coronal sections were prepared at 50 μm thickness containing the auditory field as identified by Paxino and Franklin’s mouse atlas. Sections were heat-treated using a water bath with sodium citrate buffer (pH 6) for 30 min at 80°C for antigen retrieval. To detect EdU-incorporated cells using an imaging kit (Click-iT EdU 647, Invitrogen), brain sections were washed twice with 3% BSA in PBS, permeabilized with 0.5% Triton X-100 in PBS, washed again twice with 3% BSA in PBS, then incubated with a Click-iT reaction cocktail. Next, for staining with mouse primary antibodies, sections were incubated for 1 hour in M.O.M. Mouse Ig blocking reagent (Vector) and then in mouse anti-GAD67 primary antibody (1:1000, Millipore) in the same blocking reagent. After washing with PBS, they were incubated with goat blocking solution (5% goat serum, 0.3% Triton X-100 in PBS) for 90 min and then rocked overnight with a rabbit polyclonal antibody against Ki67 in the blocking reagent at 4°C. The sections were washed with PBS and incubated with secondary antibodies (Alexa488-anti-rabbit or Cy3-anti-mouse) for 90 min at room temperature. Finally, nuclei were stained with 5 μg/ml Hoechst 33342 (Sigma) and mounted on microscope slides using a fluorescence anti-fade medium (Vector Laboratories). After imaging, fluorescence positive cells in all sections were counted.

### Western blotting

Samples from brain block tissues of auditory area cortex or cultured cells were homogenized with a lysis buffer (10 mM Tris HCl, 1 mM EDTA, 1 mM sodium orthovanadate, 1mM PMSF 2.5 mM sodium pyrophosphate, 1 mM β-glycerophosphate, protease inhibitor cocktail (Sigma), and 1% Triton X-100). Protein concentration was determined by BCA assay or micro BCA assay (Thermo). Homogenates were mixed with a 5x sample buffer: 312.5 mM Tris (pH6.8), 50% glycerol, 10% SDS, 5% β-mercaptoethanol, 0.00625% bromophenol blue. An equivalent amount of samples were loaded and separated by 10% polyacrylamide gel electrophoresis and transferred to PVDF transfer membranes (Immoblin-P, Millipore). Membranes were blocked with a blocking solution containing (5% non-fat dry milk (SACO Foods) and 5% protease-free bovine serum albumin (EQUTECH-BIO) in TBS-T (50 mM Tris-buffered saline with 0.1% (v/v) Tween-20)) for 60 min at room temperature. After washing with TBS-T, membranes were incubated with primary antibodies (mouse anti-phospho-Akt, 1:1000, CST; mouse anti-Akt, 1:1000, CST; mouse anti-β-Actin, 1:1000, rabbit anti-GAPDH, 1:1000, Sigma) diluted in same the blocking solution at 4°C overnight. Following washing with TBS-T, membranes were incubated with secondary antibodies (HRP-conjugated anti-rabbit IgG, Jackson; HRP-conjugated anti-mouse IgG, Jackson) at 1:10000 for brain tissue or at 1:1000 for cultured cells. The immunoblots were detected by chemiluminescence (ECL Prime Western blotting detection regent, BioRad) with a FAS1000 imaging system (Fujifilm, Tokyo, Japan). The optical density of protein bands was quantified using gel analysis function of NIH Image J software.

### Statistical Analysis

Values were presented as mean ± SD or mean ± SEM as indicated. Student’s two-tailed unpaired t-test or non-parametric Kolmogorov-Smirnov 2-sample test (KS test) was used to analyze statistical differences between 2 groups or among multiple groups, respectively.

## Results

### Effects of transcranial LLI on cell migration of NSPCs induced by mild ischemia

We first examined the influence of LLI on proliferated NSPCs induced by mild ischemia. Common carotid artery (CCA) was occluded for 10 min bilaterally, and 24 hours later, transcranial LLI was made to one side of cranial surface at the stereotaxically determined location of primary auditory cortex (A1) so as to avoid LLI on the contralateral side (i.e. control side; Fig 1A). TTC staining indicated that neither LLI for 60 min nor bilateral CCA occlusion for 10 min induced cell death compared with non-occlusion of sham control (Fig 1B). To determine if LLI induces cell proliferation, mice were injected with EdU (50 mg/kg mouse, i.p.) at the end of 60 min LLI and sacrificed by PFA perfusion 4 hours or 5 days after LLI (Fig 1C). Fluorescent labeling of EdU along with immunofluorescence co-staining against a proliferating cell marker Ki67 and an inhibitory neuron marker glutamic acid decarboxylase 67 (GAD67) showed that newly generated EdU positive cells that are also GAD67- and Ki67-immnopositive cells were present 4 hours after LLI (Post-LLI, Fig 1D). These cells are considered to be proliferating GABAergic cells generated after LLI and EdU injection. Such cells were found mostly in layer 1 of A1 [Control: 66.10 ± 33.30 cells/mm^3^, 4h post-LLI: 100.89 ± 30.39 cells/mm^3^ (t-test, p = 0.528)] at this time point. In addition, we could not find any EdU positive and GAD67 positive cells in non-occluded sham control (3 experiments). This result implicates that proliferation of NSPCs in the mouse cortex is promoted by mild ischemia as in the rat cortex [24].

We then looked for the effect of LLI on migration of proliferated cells. After 5 days of LLI, the number of EdU and GAD67 positive, but Ki67 negative, (EdU+/GAD+/Ki67-) cells was counted in each layer in irradiated side and control side of A1 (Fig 1E, S1 Table). The laminar distribution pattern in LLI and control group was statistically examined using the non-parametric KS 2-sample test (Fig 1F, S2 Table), which demonstrated significant difference between the two groups (n = 4, p < 0.01). The cumulative fractions from layer 1 to layer 4 showed a large difference (38.2%: 70.7% in control vs 32.6% in LLI) and those from layer 5 to layer 6 also showed an opposite difference (-38.1%: 29.3% in control vs 67.4% in LLI). Given that the total cell number in all layers combined showed no significant difference (Fig 1G, S3 Table), these data suggest that LLI induced “migration” of EdU positive cells from layer 1 to the deep layers.

### Effects of transcranial LLI on pAkt and Akt expression in mouse cortex

**Fig 2:**
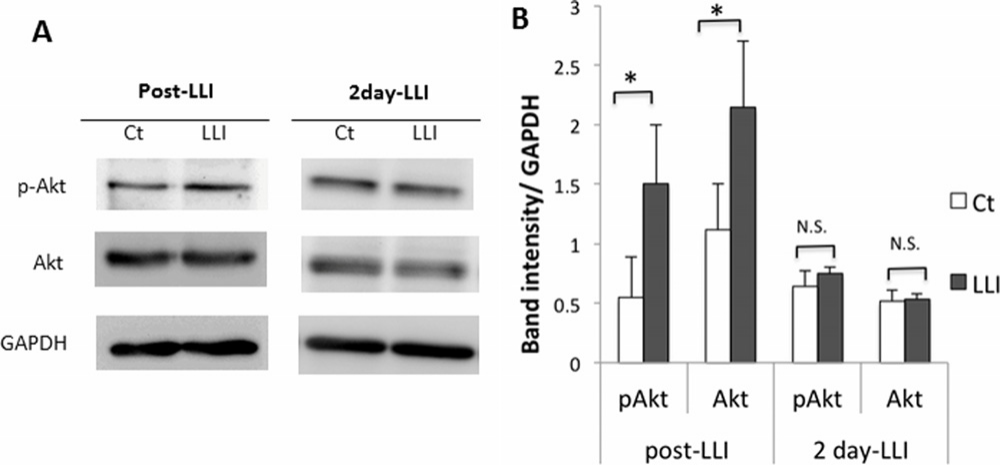
Transcranial LLI effects on pAkt and Akt expression. A: Western blot analysis of auditory cortex lysates at post-LLI and 2 days after LLI. GAPDH is a loading control. B: Quantification of blots using Image J. Band intensity was normalized to GAPDH (mean ± SD, n = 4, t-test *p < 0.05).

To find possible molecular mechanisms of LLI-induced migration of GAD67-positive NSPCs, we examined the expression of phosphorylated Akt (pAkt) and Akt. Auditory cortex treated with LLI was homogeneized at 4 hours and 2 days after LLI (Fig 2). Western blot analysis was performed for pAkt, Akt and GAPDH (Fig 2A), and their blots were quantified. When normalized to GAPDH (loading control), both pAkt and Akt expressions showed significant increase when 4 hours post-LLI was compared with non-irradiated control (Fig 2B, S4 Table), but not significant difference was seen at 2 days after LLI. These data may indicate that pAkt and Akt are upregulated soon after LLI to initiate migration process.

### LLI effect on cell proliferation of cultured NSPCs derived from E10 forebrain

**Fig 3:**
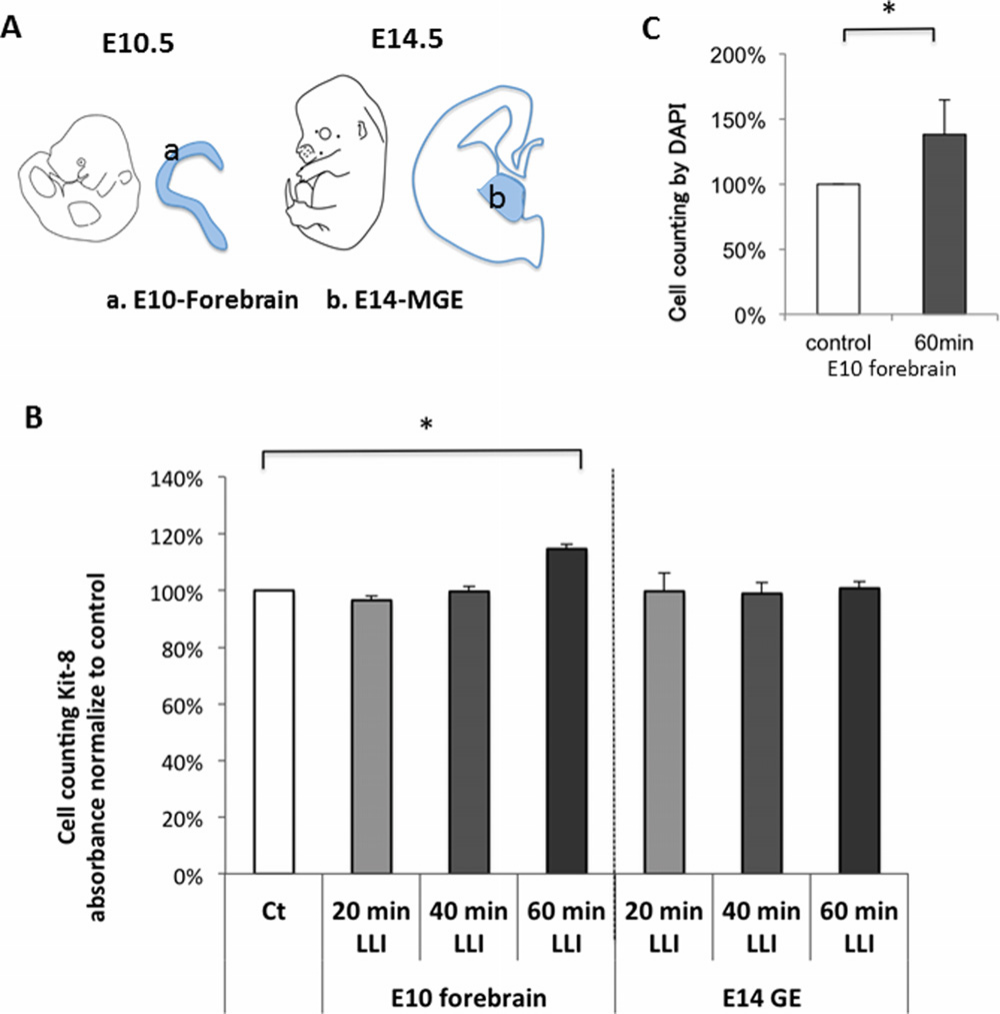
LLI effects on proliferation of NSPC cells from E10 forebrain and E14 MGE. A: Location of brain parts dissected for making neurosphere of NSPCs. “a” shows cortical wall of E10 forebrain, which generates excitatory neurons, whereas “b” shows E14 MGE, which generates GABAergic neurons. B: CCK-8 assay to show biochemically cell proliferation for different duration of LLI (mean ± SD, n = 4 for each duration, t-test, *p < 0.05). The non-irradiated group was standardized as 100%. C: Cell counting of DAPI staining to clarify the increase in cell number following the dissociation from neurospheres. LLI significantly promoted proliferation (mean ± SD, n = 5, t-test, *p < 0.05).

To examine the LLI effects on NSPCs of different types, we prepared neurospheres from E10 forebrain, which generate excitatory neurons, and those from E14 MGE, which generate inhibitory GABAergic neurons (Fig 3A). Using the CCK-8 assay, which allows biochemical determination of cell proliferation rates, we observed that 60 min LLI promoted proliferation of neurospheres derived from E10 forebrain but not derived from E14 MGE (Fig 3B, S5 Table). Additionally, LLI significantly increased the number of dissociated cells prepared from E10 forebrain compared with control as determined by DAPI staining (Fig 3C, S6 Table). These data suggest that LLI induces proliferation of NPSCs that generate excitatory neurons, but not NPSCs that become GABAergic neurons, supporting the idea that LLI induces migration, not proliferation, of GABAergic cells in A1.

### LLI effect on cell migration of cultured NSPCs derived from E14 MGE

**Fig 4:**
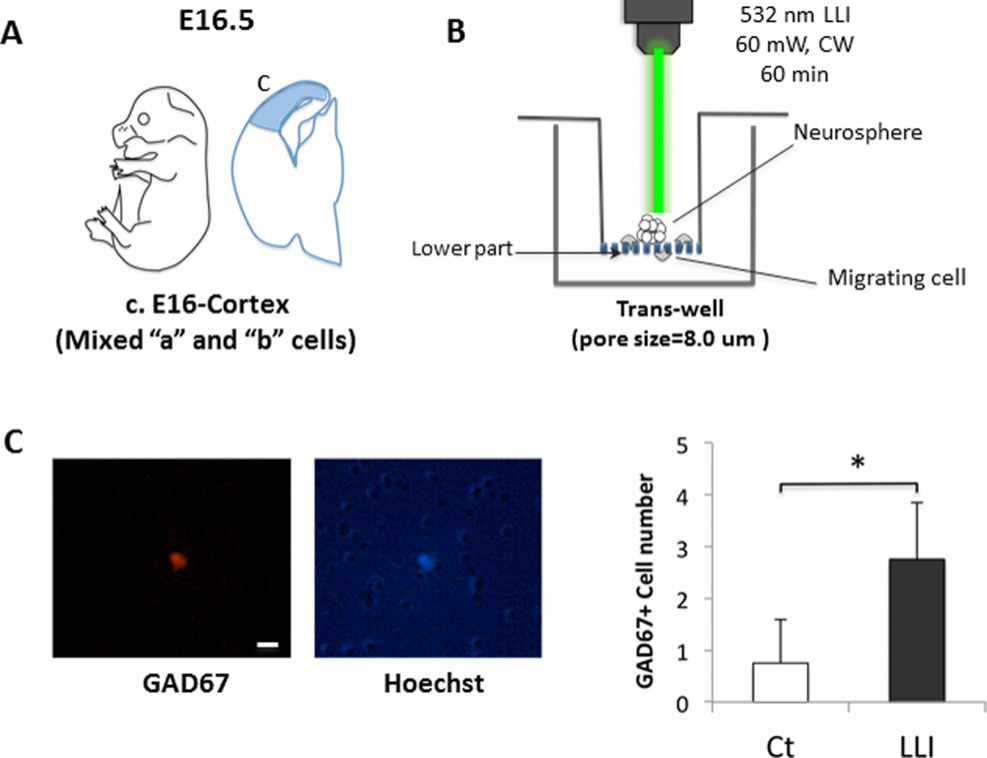
Trans-well migration experiments. A: Location of brain parts dissected for making neurospheres of NSPCs. Label “c” shows the cortex of E16, which includes NSPCs of excitatory and GABAergic neurons. B: Method of trans-well migration test. Bottom membrane of inserted trans-well has pores of 8 μm diameter. Neurospheres derived from “c” were placed on insert trans-well, and 532 nm laser was irradiated from above the media. Cells were fixed by 4% PFA at 48 h after LLI. C: A cell that moved through the pores stained by GAD67 (red) and Hochest (blue). Holes in the Hochest image are the pores of trans-well. Scale bar: 10 μm. Double positive cells were found only in the LLI experimental group (mean ± SD, n = 4, t-test *p < 0.05).

We then examined whether LLI can induce cell migration in vitro. NSPCs were collected from E16 cortex, which included excitatory and inhibitory NSPCs. Neurospheres were cultured in trans-well plates and received LLI from above (Fig 4B). At 48 h after LLI, GAD67-positive cells, but not GAD67-negative cells, were found beneath the trans-well membrane only in LLI group (Fig 4C, S7 Table). LLI increased the number of GAD67-positive cells about 3-fold. No GAD67-negative cell was found to be migrating, however. These data suggest that LLI could induce cell migration on inhibitory GABAergic neurons, which is consistent with in vivo experiments above.

### LLI effect on pAkt and Akt expression of cultured cells

**Fig 5:**
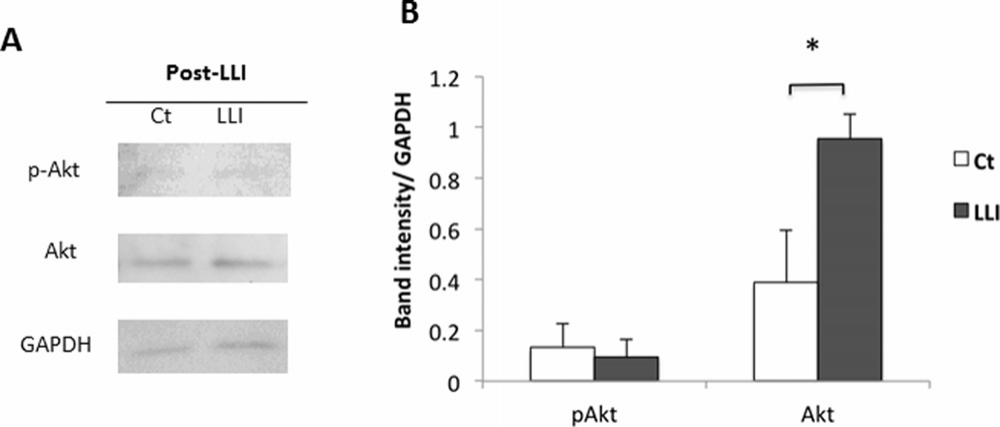
LLI effects on pAkt and Akt expression of cultured cells. A: Western blot analysis of neurosphere lysates from post-LLI (4 hours) of E10 forebrain. B: Quantification of blots. Each blot intensity was normalized to GAPDH of loading control (mean ± SD, n = 3, t-test *p < 0.05).

To test whether Akt is activated in GABAergic NSPCs, the level of phosphorylated Akt (pAkt) and Akt expressions on cultured NSPCs were examined using Western blot techniques (Fig 5A). LLI increased Akt expression but induced little changes in the phosphorylation state of Akt on NSPCs derived from the E10 forebrain (Fig 5B, S8 Table).

## Discussion

In the present study, we performed in vivo as well as in vitro experiments to reveal whether transcranial 532 nm LLI affects proliferation and migrating of GAD67-positive NSPCs in adult murine neocortex and also whether 532 nm LLI affects cultured NSPCs from embryonic mice. The in vivo experiments demonstrated that 532 nm LLI (60 mW) facilitated the migration of GABAergic neurons with a significant increase in Akt expression. Migration of GAD67-positive cells were also observed in the in vitro experiments, where 532 nm 60 min-LLI promoted the migrating of GAD67-positive NSPCs as well as the penetration beneath the trans-well membrane. These data suggest that LLI affects Akt expression and influences migration of GABAergic neurons derived from NSPCs.

It is well known that Akt plays an important role in the regulation of cellular processes that are critical for neuronal development, including gene transcription, cell proliferation, and neuronal migration. Souza et al. reported that dopamine D2 receptor activity modulates Akt signaling to promote GABAergic neurogenesis during development of zebrafish larvae [28]. Oishi et al. demonstrated that active Akt promotes differentiation of telencephalic neural precursor cells into GABA-containing, but not glutamatergic, neurons [29]. Since these studies suggest a close relationship between Akt signaling activation and developing neural cell differentiation, it is likely that our finding of LLI effects on NSPCs is based on the Akt activation.

We found that 532 nm LLI increased pAkt and Akt after 4 hours compared with non-irradiated control but not after 2 days (Fig 2). Novoselova et al. showed 632.8 nm LLI increased the interleukin-2 (IL-2) production in T cells and IL-2 concentration in blood plasma within 1 day, whereas no significant difference was seen at 2 days after the laser treatment [30]. These results suggest the effect of LLI on protein production is not permanent but temporary with a certain type of bystander effects [31].

In addition, Akt expression was enhanced in both in vivo and in vitro experiments, but pAkt was only found in vivo. In our experiments, brain tissues include not only NSPCs but also other cells, whereas cultured cells include purified NSPCs grown as neurospheres. This difference in cell environment is one possible reason for the different observations in pAkt. However, further studies will be needed to understand the different effects of LLI between in vivo and in vitro systems.

How could the 532 nm LLI affect Akt signaling pathway? The mechanism of LLI including other wavelength may depend on photoacceptors in the mitochondrial respiratory chain [32, 33] as described in detail previously [18]. However, considering that LLI induced different biological effects of cell proliferation on different cell types such as glioblastoma [16–18], skin cells [34], and NSPCs in this study, it is difficult to conclude that the regulation by mitochondrial photoacceptors is the only mechanism underlying LLI-mediated cell proliferation, differentiation and migration.

Another possibility cannot be excluded, such as direct photochemical influence on the extracellular factors that can provide molecular cues to those developmental events. There are many reports that extrinsic cues, including cell-cell interactions and secreted molecules, are key determinants of NSPC fate. Neurotrophic factors such as platelet-derived growth factor, brain-derived neurotrophic factor, and glial cell line-derived neurotrophic factor are known to promote neuronal fate, where the selective expansion of neuronal progenitors and the enhancement of the survival of neurons (or their progenitors) have been reported [35–38]. Several reports also showed that LLIs affect the release of various types of growth factors to induce beneficial effects [39–41]. Therefore, it is possible that 532 nm LLI influences the Akt signaling pathway via activation or inactivation of growth factors or cytokines.

A novel finding in this study is that 532 nm LLI promotes the migration of NSPCs into deeper layers of the neocortex (as shown in Fig 1E and 1F). Functional nature of NSPCs is unclear. The mild ischemia in adult rats facilitated proliferation, differentiation and migration [24]. Ohira et al. found that the newly generated neurons were GABAergic by GAD67 staining and that the neurons were functionally integrated into the neuronal circuitry as shown by activity-dependent c-Fos staining. Although we have not yet tried the c-Fos staining, which can detect responses to various physiological stimuli, future studies should investigate whether the migrated NSPCs induced by the LLI could be functionally integrated into cortical circuits.

The extent of LLI penetration into cortical tissues is worth discussing. The comparison to the control side of LLI in our experiments indicated direct LLI effects on the layer 1 NSPCs on the irradiated side. Although the possibility of LLI penetrating the brain from the irradiation site to the contralateral hemisphere (the control side) cannot be excluded completely, we suggest that 532 nm LLI could reach to deep layers at most, but not to the contralateral side at least 10 mm apart (Fig 1), because the penetration depth depends on LLI wavelength and LLI at short wavelength has low penetration depth [42, 43]. It was reported that the 1064 nm laser penetrates tissue (10 mm) twice as deep as the 830 nm laser (5 mm), and 10 times more than the 532 nm laser (0.8 mm) [44]. Accordingly, the transcranial approach of 532 nm LLI used here is worthy of note, because of its possible application to various experiments in vivo.

Further, our findings regarding the promotion of the GABAergic NSPC migration by 532 nm LLI might also be valuable for clinical purpose. In the case of stroke, it is well known that excessive stimulation of synaptically-activated glutamate receptors leads to signaling toward neuronal cell death [45, 46]. This excitotoxicity by tonic glutamatergic activities may be suppressed by 532 nm LLI-facilitated migration of GABAergic NSPCs before vulnerable brain regions become permanently damaged. Future studies will examine a dose-dependency of LLI (averaged power and irradiation time) to determine the most suitable transcranial approach.

## Supporting Information

S1 Table The laminar pattern of cell density of EdU and GAD67 positive, Ki67-negative cells from layer 1 to layer 6. (PDF)

S2 Table Cumulative fraction of total cells for non-parametric KS 2-sample test. (PDF)

S3 Table Total cell density of EdU and GAD 67 positive and Ki negative cells. (PDF)

S4 Table pAkt and Akt expression of auditory cortex lysates. (PDF)

S5 Table Absorbance of CCK-8 assay. (PDF)

S6 Table Cell count of DAPI staining. (PDF)

S7 Table Cell number of lower membrane on trans-well migration experiments. (PDF)

S8 Table pAkt and Akt expression of cultured cells. (PDF)
