# Supplementary material for: 532 nm Low-Power Laser Irradiation Facilitates the Migration of GABAergic Neural Stem/Progenitor Cells in Mouse Neocortex
Source: PLoS One. 2015 Apr 28;10(4):e0123833. doi: 10.1371/journal.pone.0123833 (PMC4412395; doi:10.1371/journal.pone.0123833)
Supplement: S1 Table — (PDF) [file pone.0123833.s001.pdf]

**S1 Table. EdU$^{+}$ GAD67$^{+}$ Ki67$^{-}$ cell number / mm$^{3}$**

| | Control | | | | | | | | LLI | | | | | | | |
|---|---|---|---|---|---|---|---|---|---|---|---|---|---|---|---|---|
| **mouse** | **L1** | **L2** | **L3** | **L4** | **L5a** | **L5b** | **L6a** | **L6b** | **L1** | **L2** | **L3** | **L4** | **L5a** | **L5b** | **L6a** | **L6b** |
| 1 | 0.0 | 112.9 | 0.0 | 0.0 | 56.4 | 0.0 | 0.0 | 0.0 | 0.0 | 0.0 | 51.5 | 0.0 | 103.0 | 51.5 | 0.0 | 51.5 |
| 2 | 103.0 | 53.8 | 134.4 | 26.9 | 53.8 | 0.0 | 0.0 | 0.0 | 0.0 | 146.2 | 0.0 | 29.2 | 0.0 | 58.5 | 87.7 | 0.0 |
| 3 | 90.2 | 0.0 | 0.0 | 25.7 | 0.0 | 0.0 | 0.0 | 77.1 | 21.6 | 63.9 | 95.9 | 0.0 | 32.0 | 0.0 | 0.0 | 63.9 |
| 4 | 47.4 | 0.0 | 96.3 | 72.2 | 24.1 | 48.1 | 0.0 | 72.2 | 0.0 | 0.0 | 23.0 | 0.0 | 92.1 | 115.1 | 46.0 | 138.1 |
| Mean | 60.2 | 41.7 | 57.7 | 31.2 | 33.6 | 12.0 | 0.0 | 37.3 | 5.4 | 52.5 | 42.6 | 7.3 | 56.8 | 56.3 | 33.4 | 63.4 |
| SEM | 20.2 | 23.3 | 29.6 | 13.0 | 11.6 | 10.4 | 0.0 | 18.7 | 4.7 | 30.0 | 17.9 | 6.3 | 21.2 | 20.4 | 18.3 | 24.7 |
