# Supplementary material for: 532 nm Low-Power Laser Irradiation Facilitates the Migration of GABAergic Neural Stem/Progenitor Cells in Mouse Neocortex
Source: PLoS One. 2015 Apr 28;10(4):e0123833. doi: 10.1371/journal.pone.0123833 (PMC4412395; doi:10.1371/journal.pone.0123833)
Supplement: S2 Table — (PDF) [file pone.0123833.s002.pdf]

S2. Table Cumulative fraction of total cells

	Control	LLI	Difference
L1	0.268	0.023	0.245
L2	0.366	0.186	0.180
L3	0.585	0.302	0.283
L4	0.707	0.326	0.382
L5a	0.805	0.488	0.317
L5b	0.854	0.674	0.179
L6a	0.854	0.791	0.063
L6b	1.000	1.000	0.000
D-Statistical	0.382		
D-Critical	0.344		
