# Supplementary material for: 532 nm Low-Power Laser Irradiation Facilitates the Migration of GABAergic Neural Stem/Progenitor Cells in Mouse Neocortex
Source: PLoS One. 2015 Apr 28;10(4):e0123833. doi: 10.1371/journal.pone.0123833 (PMC4412395; doi:10.1371/journal.pone.0123833)
Supplement: S3 Table — (PDF) [file pone.0123833.s003.pdf]

S3 Table. EdU$^+$ GAD67$^+$ Ki67$^-$ cell number in all layer / mm^3

mouse	Ct	LLI
1	20.2	32.4
2	48.6	38.3
3	25.3	33.9
4	45.0	38.0
Mean	34.8	35.7
SEM	6.11	1.29
