# Supplementary material for: 532 nm Low-Power Laser Irradiation Facilitates the Migration of GABAergic Neural Stem/Progenitor Cells in Mouse Neocortex
Source: PLoS One. 2015 Apr 28;10(4):e0123833. doi: 10.1371/journal.pone.0123833 (PMC4412395; doi:10.1371/journal.pone.0123833)
Supplement: S4 Table — (PDF) [file pone.0123833.s004.pdf]

S4 Table. pAkt and Akt expression of auditory cortex lysates

post-LLI

	p-Akt		Akt		GAPDH		pAkt/GAPDH		Akt/GAPDH	
	**Ct**	**LLI**	**Ct**	**LLI**	**Ct**	**LLI**	**Ct**	**LLI**	**Ct**	**LLI**
1	4127.33	4077.03	-	-	4551.67	8321.40	1.10	2.04	-	-
2	-	-	10534.93	9658.18	7483.69	3115.26	-	-	1.41	3.10
3	2665.15	11818.66	5828.71	13029.66	12125.95	7215.18	0.22	1.64	0.48	1.81
4	3968.81	8532.39	13572.58	10240.22	12275.25	5214.64	0.32	1.64	1.11	1.96
5	6736.13	10946.15	18417.12	26816.11	12617.15	15691.56	0.53	0.70	1.46	1.71
Mean	4374.35	8843.56	12088.34	14936.04	9810.74	7911.61	0.54	1.50	1.11	2.14
SD	1476.89	3003.69	4578.55	6976.33	3236.75	4276.90	0.34	0.49	0.39	0.56

2 days after LLI

	p-Akt		Akt		GAPDH		pAkt/GAPDH		Akt/GAPDH	
	**Ct**	**LLI**	**Ct**	**LLI**	**Ct**	**LLI**	**Ct**	**LLI**	**Ct**	**LLI**
1	8838.37	13953.46	8401.80	9611.61	18671.77	20232.25	0.47	0.69	0.45	0.48
2	13711.34	14607.97	8013.32	9908.32	17775.69	17866.52	0.77	0.82	0.45	0.55
3	13461.56	13285.10	12630.97	10356.12	19511.42	18016.54	0.53	0.40	0.33	0.71
Mean	12003.76	13948.84	9682.03	9958.68	18652.96	18705.11	0.59	0.63	0.41	0.58
SD	2240.59	540.07	2091.24	306.02	708.73	1081.59	0.13	0.18	0.05	0.10
