# Supplementary material for: 532 nm Low-Power Laser Irradiation Facilitates the Migration of GABAergic Neural Stem/Progenitor Cells in Mouse Neocortex
Source: PLoS One. 2015 Apr 28;10(4):e0123833. doi: 10.1371/journal.pone.0123833 (PMC4412395; doi:10.1371/journal.pone.0123833)
Supplement: S5 Table — (PDF) [file pone.0123833.s005.pdf]

S5 Table.Absorbance of cell count kit-8 assay

	Absorbance of CCK-8				Normalized (LLI/Ct)		
**E10-FB**	**Control**	**20 min LLI**	**40 min LLI**	**60 min LLI**	**20 min LLI**	**40 min LLI**	**60 min LLI**
1	0.178	0.174	0.182	0.208	0.978	1.022	1.169
2	0.178	0.173	0.173	0.202	0.975	0.975	1.138
3	0.206	0.194	0.204	0.233	0.944	0.993	1.134
Mean	0.187	0.180	0.186	0.214	0.965	0.997	1.147
SD	0.013	0.010	0.013	0.013	0.015	0.020	0.015

	Absorbance of CCK-8				Normalized (LLI/Ct)		
**E14-MGE**	**Control**	**20 min LLI**	**40 min LLI**	**60 min LLI**	**20 min LLI**	**40 min LLI**	**60 min LLI**
1	0.150	0.162	0.157	0.158	1.080	1.047	1.053
2	0.154	0.162	0.153	0.154	1.055	0.997	1.003
3	0.158	0.145	0.146	0.159	0.921	0.927	1.010
4	0.157	0.146	0.152	0.156	0.930	0.968	0.994
5	0.146	0.146	0.147	0.143	1.000	1.007	0.979
Mean	0.153	0.152	0.151	0.154	0.997	0.989	1.008
SD	0.004	0.008	0.004	0.006	0.064	0.040	0.025
