# Supplementary material for: 532 nm Low-Power Laser Irradiation Facilitates the Migration of GABAergic Neural Stem/Progenitor Cells in Mouse Neocortex
Source: PLoS One. 2015 Apr 28;10(4):e0123833. doi: 10.1371/journal.pone.0123833 (PMC4412395; doi:10.1371/journal.pone.0123833)
Supplement: S6 Table — (PDF) [file pone.0123833.s006.pdf]

S6 Table. Cell count of DAPI staining

	Ct	LLI	LLI/Ct
1	13	15	1.489
2	94	140	1.380
3	152	209	1.017
4	89	90	1.810
5	79	143	1.200
Mean	85.1	119.4	1.379
SD	44.3	64.5	0.269
