# Supplementary material for: 532 nm Low-Power Laser Irradiation Facilitates the Migration of GABAergic Neural Stem/Progenitor Cells in Mouse Neocortex
Source: PLoS One. 2015 Apr 28;10(4):e0123833. doi: 10.1371/journal.pone.0123833 (PMC4412395; doi:10.1371/journal.pone.0123833)
Supplement: S7 Table — (PDF) [file pone.0123833.s007.pdf]

S7 Table. Cell number of Lower membrane

	Ct	LLI
1	0.0	1.0
2	0.0	3.0
3	2.0	4.0
4	1.0	3.0
Mean	0.750	2.750
SD	0.829	1.090
