# Supplementary material for: 532 nm Low-Power Laser Irradiation Facilitates the Migration of GABAergic Neural Stem/Progenitor Cells in Mouse Neocortex
Source: PLoS One. 2015 Apr 28;10(4):e0123833. doi: 10.1371/journal.pone.0123833 (PMC4412395; doi:10.1371/journal.pone.0123833)
Supplement: S8 Table — (PDF) [file pone.0123833.s008.pdf]

S8 Table. pAkt and Akt expression of cultured cells

	p-Akt		Akt		GAPDH		p-Akt/GAPDH		p-Akt/GAPDH	
	**Ct**	**LLI**	**Ct**	**LLI**	**Ct**	**LLI**	**Ct**	**LLI**	**Ct**	**LLI**
1	837.4	861.9	2735.7	4912.8	4171.1	5936.3	0.201	0.145	0.656	0.828
2	0.0	0.0	603.9	1973.5	1725.5	2048.9	0.000	0.000	0.350	0.963
3	389.8	412.7	330.2	3144.0	2010.9	2941.7	0.194	0.140	0.164	1.069
Mean	409.1	424.9	1223.3	3343.4	2635.9	3642.3	0.132	0.095	0.390	0.953
SD	342.1	352.0	1075.3	1208.2	1091.8	1662.5	0.093	0.067	0.203	0.099
